# The Electrical and Thermal Transport Properties of La-Doped SrTiO_3_ with Sc_2_O_3_ Composite

**DOI:** 10.3390/ma14216279

**Published:** 2021-10-21

**Authors:** Kai Guo, Fan Yang, Tianyao Weng, Jianguo Chen, Jiye Zhang, Jun Luo, Han Li, Guanghui Rao, Jingtai Zhao

**Affiliations:** 1 School of Physics and Materials Science, Guangzhou University, Guangzhou 510006, China; lihan@gzhu.edu.cn; 2 School of Materials Science and Engineering, Shanghai University, 99 Shangda Road, Shanghai 200444, China; yangfan0720@shu.edu.cn (F.Y.); ty-weng@shu.edu.cn (T.W.); kpfocus@shu.edu.cn (J.C.); jychang@shu.edu.cn (J.Z.); junluo@shu.edu.cn (J.L.); 3 Materials Genome Institute, Shanghai University, 99 Shangda Road, Shanghai 200444, China; 4 School of Materials Science and Engineering, Guilin University of Electronic Technology, Guilin 541004, China; rgh@guet.edu.cn; 5 Guangxi Key Laboratory of Information Materials, Guilin University of Electronic Technology, Guilin 541004, China

**Keywords:** strontium titanate, rare earth doping, composite, thermal expansion, lattice thermal conductivity

## Abstract

Donor-doped strontium titanate (SrTiO_3_) is one of the most promising n-type oxide thermoelectric materials. Routine doping of La at Sr site can change the charge scattering mechanism, and meanwhile can significantly increase the power factor in the temperature range of 423–773 K. In addition, the introduction of Sc partially substitutes Sr, thus further increasing the electron concentration and optimizing the electrical transport properties. Moreover, the excess Sc in the form of Sc_2_O_3_ composite suppresses multifrequency phonon transport, leading to low thermal conductivity of *κ* = 3.78 W·m^−1^·K^−1^ at 773 K for sample Sr_0.88_La_0.06_Sc_0.06_TiO_3_ with the highest doping content. Thus, the thermoelectric performance of SrTiO_3_ can be significantly enhanced by synergistic optimization of electrical transport and thermal transport properties via cation doping and composite engineering.

## 1. Introduction

With the sustainable development of global industrialization, the demand for energy is rapidly growing in recent years, which promotes researchers to explore clean and renewable energy technology. Thermoelectric (TE) materials, enabling the direct interconversion between heat and electricity based on the Seebeck effect and the Peltier effect, would play important role in the energy depletion [1,2,3]. The conversion efficiency of TE materials is essentially determined by the dimensionless figure of merit *ZT* = *σS*^2^*T*/(*κ_lat_* + *κ_e_*). where *σ*, *S*, *T*, *κ_lat_*, and *κ_e_* represent the electrical conductivity, Seebeck coefficient, absolute temperature, the lattice and electronic components of thermal conductivity *κ_tot_*, respectively [4]. Accordingly, high thermoelectric properties require synergistic optimization of electrical and thermal transport properties, and thus lattice softening [5], nanostructure engineering [6,7], band convergence [8,9,10], multiscale phonon scattering including dislocation engineering [11,12], point defect and grain boundary scattering [13,14], have been proposed and developed in these years.

Due to the low-cost, excellent thermal stability, environmental compatibility, and unique oxidation-proof features at high temperatures, transition metal oxides such as Na*_x_*CoO_2_ [15,16], and Ca_3_Co_4_O_9_ [17,18,19] are suitable for p-type thermoelectric candidates. Especially, their significantly high thermal stability allows maintaining large temperature differences (Δ*T*) in thermoelectric devices, making them possible to achieve high output power [20,21]. However, the poor electrical conductivity restricts the power factor (*PF* = *σS*^2^) and thermoelectric figure of merit *ZT*. Therefore, many researches have been focusing on the strategies for optimizing the electrical transport properties. For example, a *PF* as high as 18.92 μW·cm^−1^ K^−2^ at 1100 K in Na*_x_*CoO_2_ can be realized via Ag composite with large electron density of ~10^21^·cm^−3^ [22].

For n-type oxide thermoelectric materials, strontium titanate (STO) undergoing donor-doping has obtained much attention as a result of their promising thermoelectric properties [23,24]. The band structure calculations reveal that there are heavy and light bands around the Fermi level contributing to the electron transport in SrTiO_3_, favoring large Seebeck coefficients [25]. In this situation, large power factors of 28–36 μW·cm^−1^ K^−2^ at room temperature has been achieved in n-type Sr_1*-x*_La*_x_*TiO_3_ single crystal with relatively high carrier concentrations of (0.2–2) × 10^21^ cm^−3^ [26]. However, the thermoelectric performance can be further boosted with reduced thermal conductivities. In context of the lattice thermal conductivity *κ_lat_* contributing 75–100% of the total thermal conductivity, suppression of phonon transport would enable the optimization of thermoelectric performance in perovskite titanate thermoelectrics (ABO_3_) [27]. The simple and effective strategy is to introduce point defects by disordering A site to strengthen the phonon scattering. It is reported that doping ions with a smaller ion radius at the A site can reduce thermal conductivity well, while doping ions with a closed radius with Sr can significantly improve electrical transport performance [28,29].

In this work, La doping and Sc_2_O_3_ composite have been utilized for the synergistic optimization of electrical and thermal transport properties. Substitution Sr with trivalent La aims to increase the electrical conductivity of SrTiO_3_, while compositing Sc_2_O_3_ is expected to reduce the thermal conductivity. The power factor reaches 9.41 μW·cm^−1^·K^−2^ at 517 K. In addition, point defect induced the stress and mass fluctuation favor for the enlargement of expansion coefficients and reduction of lattice thermal conductivity. As a result, the *ZT* = 0.143 has been obtained for the sample Sr_0.88_Sc_0.06_La_0.06_TiO_3_ at 773 K.

## 2. Materials and Methods

### 2.1. Sample Preparation

Undoped and doped strontium titanate powders were prepared by solid state reaction method, using SrCO_3_ (99.8%), TiO_2_ (99.8%), La_2_O_3_ (99.9%), and Sc_2_O_3_ (99.9%) as raw materials. These powers were weighted according to the stoichiometric ratio Sr_1*-x-y*_Sc*_x_*La*_y_*TiO_3_ (*x* = 0, 0.04, 0.06; *y* = 0, 0.06), and mixed via ball milling at a speed of 200 r/min for 48 h with stainless steel pots and zirconia balls. The as-obtained mixtures were cold-pressed into tablets with ϕ10 mm × 2 mm, which were then placed in a muffle furnace for annealing at 1573 K for 6 h in air. The as-annealed samples were ground into fine powders by ball milling again with 500 r/min for 12 h. Finally, dense ceramic samples (ϕ10 mm × 2 mm) were prepared by spark plasma sintering (SPS) with graphite dies under 1473 K and 30 MPa for 5 min.

### 2.2. Phase and Microstructure Characterization

The phase purity of the as-prepared samples was examined by powder X-ray diffraction (PXRD, Rigaku, Japan, Cu K*α* radiation, *λ* = 1.541854 Å, 20° < 2θ < 80°, step width 0.02°) at room temperature. The lattice parameters were calculated using the software of WinCSD (version 4.19, L. Akselrud. Kyiv, Ukraine) [30]. The microstructure and composition were characterized by scanning electron microscope (SEM; ZEISS Gemini 300, Jena, Germany), equipped with energy-dispersive X-ray spectroscopy (EDX), which was performed at the accelerating voltage of 15 kV (Oxford X-MAX, Oxford, UK). The average grain sizes were examined from the observed microstructure by image analysis using the Image-Pro program (Plus 6.0, 2018, Media Cybernetics, MD, USA) [31].

### 2.3. Thermoelectric Performance Measurements

The Seebeck coefficient and electrical conductivity of the samples were simultaneously measured using a ZEM-3 instrument (ULVAC-RIKO, Kanagawa, Japan) under helium atmosphere from room temperature to 773 K. The room temperature Hall coefficient (*R*_H_), Hall carrier concentration (*n*_H_), and Hall mobility (*μ*_H_) were collected with a Hall effect test system (Lake Shore 8400, Westerville, OH, USA) using the four-probe van der Pauw method under a reversible magnetic field of 0.9 T. The thermal expansion coefficients were obtained from 500 K to 800 K by a thermomechanical analyzer (NETZSCH, TMA 402F3, Selb, Germany). The thermal conductivity can be calculated according to the equation *κ* = *C*_p_*λd*, where *C*_p_ is the specific heat capacity, *λ* is the thermal diffusivity, and *d* is the density. A laser flash diffusivity (NETZSCH, LFA467, Selb, Germany) was used to measure *λ* of a tablet sample with a diameter of 10 mm and a typical thickness of 1–2 mm. Prior to the measurement, the samples were coated with a thin graphite layer to minimize the error of material emissivity. The specific heat capacity (*C*_p_) was determined by the experimental measurement with a thermal analyzer (NETZSCH, STA 449F3, Selb, Germany) using sapphire as reference sample. The density *d* was measured at room temperature by applying the Archimedes method with ethanol as the immersion liquid.

## 3. Results and Discussion

**Figure 1:**
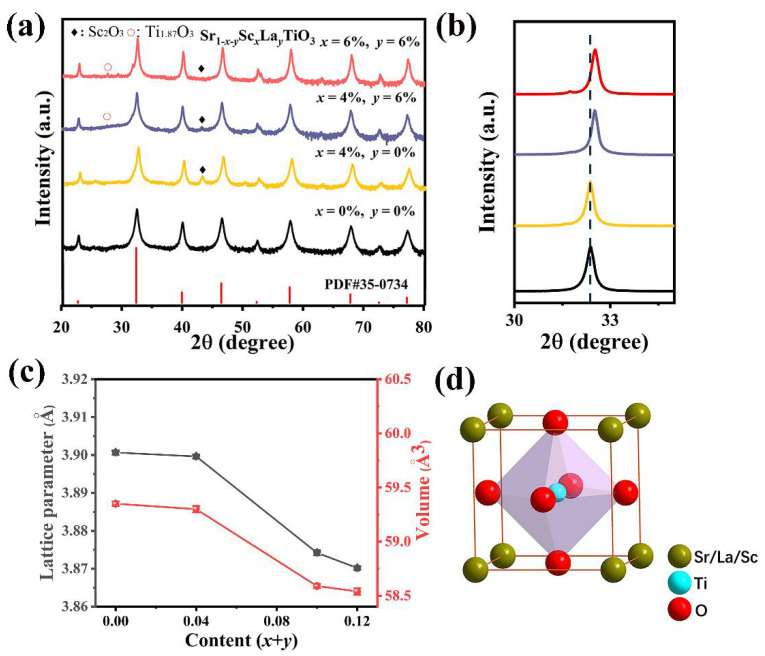
(**a**) PXRD patterns of Sr_1-*x-y*_Sc*_x_*La*_y_*TiO_3_ (*x* = 0, 0.04, 0.06; *y* = 0, 0.06) ceramics, (**b**) the enlarged peak in the vicinity of 33°, (**c**) lattice parameter and volume of Sr_1*-x-y*_Sc*_x_*La*_y_*TiO_3_ (*x* = 0, 0.04, 0.06; *y* = 0, 0.06) ceramics as function of the doping contents, (**d**) crystal structure diagram of SrTiO_3_.

The PXRD results of Sr_1-*x-y*_Sc*_x_*La*_y_*TiO_3_ (*x* = 0, 0.04, 0.06; *y* = 0, 0.06) samples are shown in Figure 1a. Almost all diffractions are well consistent with cubic perovskite structure (Figure 1d.) despite the fact that a small amount of impurity phase identified as Sc_2_O_3_ and Ti_1.87_O_3_ can be tracked. Figure 1b displays the diffractions around 33°, which are basically unchanged for single-doped samples. This can be understood from the low solid solubility of Sc in SrTiO_3_ due to the large difference in ionic radius [32]. As a matter of fact, Sc is usually treated as dopant for Ti in SrTiO_3_ to tune the physical properties [33]. High-angle shift is observed for La/Sc co-doped samples, demonstrating that La can successfully substitute Sr since the ionic size of La^3+^ (1.36 Å, 12-coordination) is slightly smaller than that of Sr^2+^ (1.44 Å, 12-coordination) [34]. The dependences of lattice parameters on the doping contents verify the conclusion presented in Figure 1c. The lattice parameters are constant with single Sc doping, and get smaller when La substitutes Sr in SrTiO_3_.

**Figure 2:**
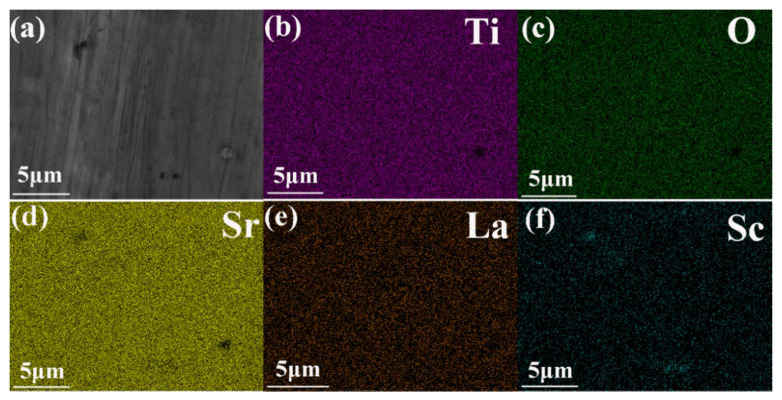
(**a**) SEM images of typical sample Sr_0.9_Sc_0.04_La_0.06_TiO_3_; (**b**–**f**) the corresponding elemental distribution for Ti, O, Sr, La, and Sc.

Figure 2a presents the SEM image of the surface for the co-doped sample Sr_0.9_Sc_0.04_La_0.06_TiO_3_. The element distributions of Sr_0.9_Sc_0.04_La_0.06_TiO_3_ are basically homogeneous (Figure 2b–f), suggesting La and partial Sc can be dissolved into the matrix. However, a small amount of Sc enrichment area can also be observed, indicating that the low solution limit of Sc, which is well in agreement with the XRD results. Table 1 shows the real compositions of Sr_1*-x-y*_Sc*_x_*La*_y_*TiO_3_ (*x* = 0, 0.04, 0.06; *y* = 0, 0.06) detected by EDS, close to the nominal compositions designed in this work.

The average grain sizes were measured from SEM images by image analysis using the Image-Pro program [31], as can be seen in Table 2. With the increase of doping amount, the average grain sizes are almost the same, ranging from 1.35 μm to 1.87 μm. It is reported that the mean free paths of electron and phonon in SrTiO_3_ are about 1 nm and 2 nm, respectively [35,36]. Thus, the large grains would not result in the essential difference in electron and phonon transport for samples Sr_1-*x-y*_Sc*_x_*La*_y_*TiO_3_ (*x* = 0, 0.04, 0.06; *y* = 0, 0.06). The measured densities are very close to the ideal value of single crystal SrTiO_3_, suggesting dense feature for bulk samples.

**Figure 3:**
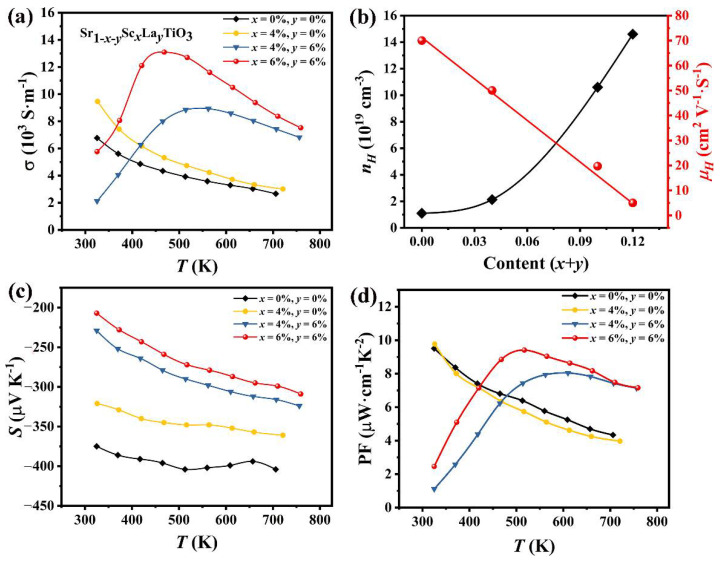
(**a**) Temperature-dependent electrical conductivity (*σ*). (**b**) Hall carrier concentration (*n*_H_) and mobility (*μ*_H_) as a function of the Sc/La contents at room temperature. (**c**) Temperature-dependent Seebeck coefficient (*S*), and (**d**) power factor (*PF*).

Figure 3 shows the temperature-dependence of the electrical transport properties for Sr_1-*x-*y_Sc*_x_*La*_y_*TiO_3_ (*x* = 0, 0.04, 0.06; *y* = 0, 0.06) ceramics. As can be seen in Figure 3a, the pristine SrTiO_3_ and single Sc-doped samples exhibit metal-like conductive behaviors. Meanwhile, the electrical conductivity of Sr_0.96_Sc_0.04_TiO_3_ increases slightly in comparison with undoped SrTiO_3_, confirming finite substitution of Sc^3+^ for Sr^2+^, which introduces extra electrons and increases the electron concentration (Figure 3b). La-doping enhances the electrical conductivity of SrTiO_3_ significantly, and the conduction behaviors transform from metal to semiconductor before 468 K. The electrical conductivity increases at low temperatures, while decreases at high temperatures with increasing temperature, which are consistent with the results reported in the literatures [23,37]. However, the values of electrical conductivity are lower than the data reported in the literatures, which is probably ascribed from the Sr vacancy since Sc hardly substitutes Sr.

At room temperature, Sr_0.96_Sc_0.04_TiO_3_ sample has the largest electrical conductivity because of its high mobility (Figure 3b). For La/Sc co-doped samples, the carrier mobilities μ_H_ descend, resulting in the low electrical conductivity. At high temperature, the electrical conductivity of all samples monotonically increase with the rise of the doping concentration, which is derived from the donor doping effect. Figure 3c shows the Seebeck coefficients of Sr_1-*x-*y_Sc*_x_*La*_y_*TiO_3_ (*x* = 0, 0.04, 0.06; *y* = 0, 0.06) depending on the temperature. The negative S in all measurements indicates that Sr_1-x-y_Sc_x_La_y_TiO_3_ ceramics are n-type semiconductors. The absolute values of Seebeck coefficient for Sr_1-x-y_Sc_x_La_y_TiO_3_ increases as the temperature rises, and doping suppresses the Seebeck coefficient since it is inversely proportional to the carrier concentration as follows: S~[π/(3n)]^2/3^ m × T (m is the electron effective mass) [38]. The power factors *PF* are calculated from *σS^2^* and presented in Figure 3d. The undoped and single-doped Sc samples have the largest power factor at room temperature (9.8 μW·cm^−1^·K^−^^2^ at 320 K). For the co-doped samples, the peak values shift to the high temperature and *PF* = 9.41 μW·cm^−1^·K^−2^ has been achieved for Sr_0.88_Sc_0.06_La_0.06_TiO_3_.

**Figure 4:**
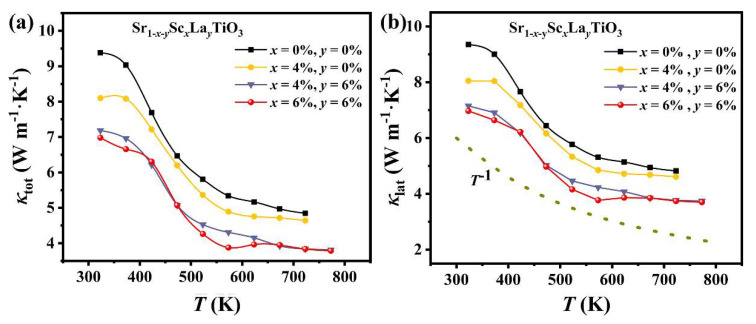
The temperature dependences of (**a**) the total thermal conductivity (*κ_tot_*), and (**b**) the lattice thermal conductivity (*κ_lat_*). The dotted line indicates that the temperature-dependent lattice thermal conductivity satisfies *T*^−1^ relation when phonon scattering dominates.

The thermal expansion coefficients (*α_V_*) ranging from 500–800 K for these samples are shown in Table 2. With the increase of the doping amount at the Sr site, this parameter increases monotonically, which leads to the reduction in the lattice thermal conductivity (Figure 4b). The lattice thermal conductivity is inversely proportional to the absolute value of the average volumetric thermal expansion coefficients, expressed by Grüneisen’s law and the Slack phonon model as follows:

(1) $$\alpha_{V}=\frac{\beta_{T}}{V}\gamma C$$

(2) $$\kappa_{lat}=A\frac{\bar{M}\theta_{d}^{3}\delta }{\gamma^{2}n^{2/3}T}$$

where *A* is a constant, $\bar{M}$ is the average atomic mass, *n* is the number of atoms per unit cell, *δ^3^* is the volume per atom, *T* is the absolute temperature, *γ* is the average Grüneisen parameter for the acoustic branches, and *θ_d_* is the Debye temperature, *β_T_* is the isothermal compressibility, *C* is the heat capacity, and *V* is the molar volume. From Equations (1) [39] and (2) [40], *α_V_* is directly proportional to the Grüneisen parameter *γ* under certain conditions. On the other hand, *γ* is usually the parameter that characterizes the strength of anharmonic inversely proportional to *κ_lat_*.

Lattice thermal conductivity *κ_lat_* is calculated by subtracting *κ_e_* from *κ_tot_*, and the electronic contribution *κ_e_* is estimated according to the Wiedemann-Franz law (*κ_e_* = *LσT*) with the Lorentz umber (*L*) estimated from the single parabolic band (SPB) model [38]. The contribution of *κ_e_* to the total thermal conductivity *κ_tot_* is too low, which can be negligible in this work. Thus, the varieties of *κ_tot_* and *κ_lat_* on the temperature are basically identified (Figure 4a,b). The pristine SrTiO_3_ exhibits a high thermal conductivity at room temperature, reaching 9.38 W·m^−1^·K^−1^. The relatively high thermal conductivity restricts figure of merit *ZT*, and additional scattering mechanism should be introduced to lower the lattice thermal conductivity. Figure 4b displays the lattice thermal conductivity decreases with increasing the temperature which basically conforms to the relationship of *T*^−1^, indicating that phonon scattering is dominant at high temperatures.

The lattice thermal conductivity drops sharply with La and Sc doping in SrTiO_3_. Such a significant reduction mainly ascribes to the point defect scattering due to the mass fluctuation and strain fluctuations (described by disorder scattering factors $\Gamma_{M}$ and $\Gamma_{S}$, respectively) between La/Sc and Sr, and thereby giving rise to the reduction of the lattice thermal conductivity at room temperature. The $\tau_{PD}^{-1}$ (phonon-point-defect scattering) and disorder scattering factors can be obtained through [41]:

(3) $$\tau_{PD}^{-1}=\tau_{S}^{-1}+\tau_{M}^{-1}=\frac{V\omega^{4}}{4\pi v_{s}^{3}}\left(\Gamma_{S}+\Gamma_{M}\right)$$

(4) $$\Gamma =\Gamma_{S}+\Gamma_{M}$$

(5) $$\Gamma_{S}=\frac{\sum_{i=1}^{n}c_{i}{\left(\frac{\bar{M_{i}}}{\overset{̿}{M}}\right)}^{2}f_{i}^{1}f_{i}^{2}\epsilon_{i}{\left(\frac{r_{i}^{1}-r_{i}^{2}}{{\bar{r}}_{i}}\right)}^{2}}{\sum_{i=1}^{n}c_{i}}$$

(6) $$\Gamma_{M}=\frac{\sum_{i=1}^{n}c_{i}{\left(\frac{\bar{M_{i}}}{\overset{̿}{M}}\right)}^{2}f_{i}^{1}f_{i}^{2}{\left(\frac{M_{i}^{1}-M_{i}^{2}}{{\bar{M}}_{i}}\right)}^{2}}{\sum_{i=1}^{n}c_{i}}$$

where ω is the phonon frequency, and *v_s_* is the sound speed. The disorder Γ is related to both mass fluctuation scattering $\Gamma_{M}$ and strain field $\Gamma_{S}$. *c_i_* is the relative degeneracy of the site, *f_i_* is the fractional occupation, $\bar{M_{i}}$ and ${\bar{r}}_{i}$ are the average mass and radii of element, respectively, and $\bar{M}$ is the average mass [42]. The difference in the ionic radius between Sr and rare earth elements leads to high distortion into the lattice and thus reduces the lattice thermal conductivity. In addition, Sc_2_O_3_ as a composite phase, plays a role in low-frequency phonon scattering, favoring the reduction of lattice thermal conductivity. The total thermal conductivity of the sample Sr_0.88_Sc_0.06_La_0.06_TiO_3_ significantly reduced to 6.97 W·m^−1^·K^−1^ at room temperature, which is 25.6% lower than the pristine sample.

**Figure 5:**
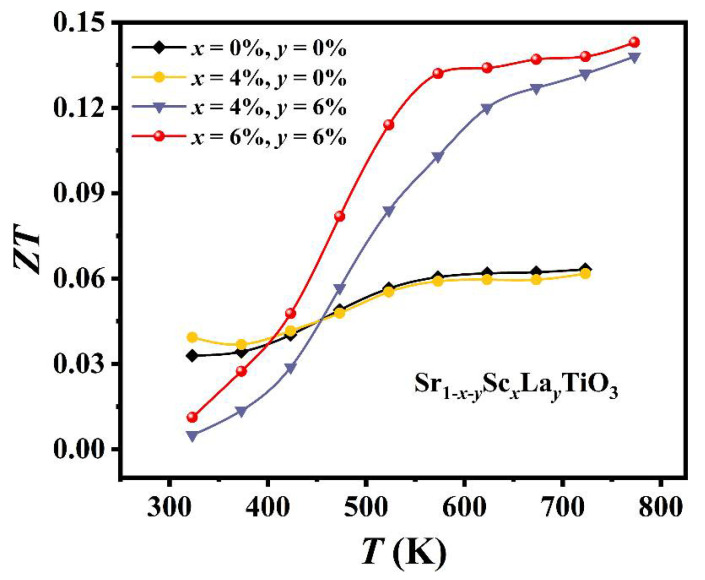
Temperature-dependence of the figure-of-merit *ZT_._* for samples Sr_1*-x-y*_Sc*_x_*La*_y_*TiO_3_ (*x* = 0, 0.04, 0.06; *y* = 0, 0.06).

Figure 5 plots the *ZT* values as a function of temperature from 323 K to 773 K. The *ZT* values increase with the rise of the temperature, and the largest *ZT* = 0.143 at 773 K has been achieved for composition Sr_0.88_Sc_0.06_La_0.06_TiO_3_. In comparison with the value reported in literature with single La doping at the same temperature (*ZT*~0.2) [43], the thermoelectric performance is uncompetitive in this work. However, the composite engineering turns out to be an effective route to optimize the electrical and thermal transport properties of thermoelectric materials.

## 4. Conclusions

The polycrystalline bulk Sr_1-*x*-*y*_Sc*_x_*La*_y_*TiO_3_ (*x* = 0, 0.04, 0.06; *y* = 0, 0.06) samples with promising thermal stability were synthesized by solid-state reaction method and spark plasma sintering. The introduction of trivalent La increases the electron concentration and improves the electrical conductivity. Sc hardly substitutes Sr as a result of the low solid solution, and it distributes in the sample in the form of Sc_2_O_3_, which favors for the reduction of lattice thermal conductivity due to the low-frequency phonon scattering. Therefore, the total thermal conductivity decreases from 9.38 W·m^−1^·K^−1^ for pristine SrTiO_3_ to 6.97 W·m^−1^·K^−1^ for Sr_0.88_Sc_0.06_La_0.06_TiO_3_ at room temperature. The enhanced *ZT* value of 0.143 was achieved at 773 K in nominal sample Sr_0.88_Sc_0.06_La_0.06_TiO_3_.

## Figures and Tables

**Table 1:** The real compositions of Sr_1*-x-y*_Sc*_x_*La*_y_*TiO_3_ (*x* = 0, 0.04, 0.06; *y* = 0, 0.06) detected by EDS.

Nominal Comp.	Ti (%)	O (%)	Sr (%)	La (%)	Sc (%)	Real Comp.
SrTiO_3_	20.01 ± 0.27	60.04 ± 0.12	19.95 ± 0.25	-	-	SrTi_0.997_O_3_
Sr_0.96_Sc_0.04_TiO_3_	20.03 ± 0.16	60.05 ± 0.13	19.20 ± 0.15	-	0.72 ± 0.28	Sr_0.959_Sc_0.036_Ti_1.001_O_3_
Sr_0.9_Sc_0.04_La_0.06_TiO_3_	19.88 ± 0.21	60.18 ± 0.31	17.98 ± 0.19	1.14 ± 0.09	0.82 ± 0.32	Sr_0.897_Sc_0.040_La_0.058_Ti_0.99_O_3_
Sr_0.88_Sc_0.06_La_0.06_TiO_3_	20.02 ± 0.18	59.92 ± 0.21	17.69 ± 0.20	1.21 ± 0.06	1.16 ± 0.21	Sr_0.885_Sc_0.058_La_0.061_Ti_1.002_O_3_

**Table 2:** Average grain sizes, densities, and thermal expansion coefficients of samples Sr_1*-x-y*_Sc*_x_*La*_y_*TiO_3_ (*x* = 0, 0.04, 0.06; *y* = 0, 0.06).

Sr_1*-x-y*_Sc*_x_*La*_y_*TiO_3_	Average Grain Size (μm)	Real Density (g cm^−3^)	Thermal Expansion Coefficients (10^−5^ K^−1^, 500–800 K)
SrTiO_3_	1.77 ± 0.07	5.105	1.00 ± 0.01
Sr_0.96_Sc_0.04_TiO_3_	1.87 ± 0.01	4.997	1.01 ± 0.01
Sr_0.9_Sc_0.04_La_0.06_TiO_3_	1.35 ± 0.07	5.124	1.02 ± 0.01
Sr_0.88_Sc_0.06_La_0.06_TiO_3_	1.35 ± 0.05	5.134	1.08 ± 0.01

## Data Availability

All the data is available within the manuscript.
